# Resilience after 3/11: structural brain changes 1 year after the Japanese earthquake

**DOI:** 10.1038/mp.2014.28

**Published:** 2014-04-29

**Authors:** A Sekiguchi, Y Kotozaki, M Sugiura, R Nouchi, H Takeuchi, S Hanawa, S Nakagawa, C M Miyauchi, T Araki, A Sakuma, Y Taki, R Kawashima

**Affiliations:** 1 Division of Medical Neuroimage Analysis, Department of Community Medical Supports, Tohoku Medical Megabank Organization, Tohoku University, Sendai, Japan; 2 Department of Functional Brain Imaging, Institute of Development, Aging and Cancer (IDAC), Tohoku University, Sendai, Japan; 3 Department of Advanced Brain Science, Smart Ageing International Research Center, IDAC, Tohoku University, Sendai, Japan; 4 International Research Institute of Disaster Science, Tohoku University, Sendai, Japan; 5 Division of Developmental Cognitive Neuroscience, IDAC, Tohoku University, Sendai, Japan; 6 Graduate Schools for Law and Politics, The University of Tokyo, Tokyo, Japan; 7 Department of Psychiatry, Tohoku University Graduate School of Medicine, Sendai, Japan; 8 Department of Nuclear Medicine and Radiology, Institute of Development, Aging and Cancer, Tohoku University, Sendai, Japan

Stressful events can have both short- and long-term effects on the brain.^1,2^ A recent investigation by our lab identified regional grey matter volume (rGMV) changes in people in the months following the Japanese earthquake.^3^ These findings indicated that smaller anterior cingulate cortex volume was a preexisting vulnerability factor for posttraumatic stress disorder (PTSD) symptoms and that decreased volume of the orbitofrontal cortex (OFC) was a result of these acquired symptoms.^3^ These types of symptoms were regarded as manifestations of the short-term effects of post-earthquake stress. However, the long-lasting effects of stressful events on brain structures remain unclear. Thus, this study examined the 1-year prognoses of subjects after a stressful event to clarify the long-term effects of stress on structural brain changes.

Of the 42 subjects included in our previous study,^3^ 37 subjects (male/female (M/F)=28/9, age=21.0±1.6 years) were recruited for a third time, and their structural magnetic resonance imaging (MRI) scans were evaluated 1 year after the earthquake. The optimized voxel-based morphometry (VBM) method for a brain structural data set (for greater detail, see Sekiguchi *et al.*,^3^) was applied, and rGMVs from before (Pre), soon after (Post) and at the 1-year follow-up (Follow-up) of the earthquake were compared using conjunction analyses. In addition, we also assessed the subjects' psychological characteristics, including anxiety, depression, posttraumatic growth and self-esteem. Furthermore, we collected longitudinal brain structural MRI data from 11 normal controls (M/F=7/4, age=20.2±1.0 years) obtained on at least two occasions before the earthquake (see Supplementary Methods for additional details).

At 1 year after the earthquake, none of the subjects in this study had developed clinical PTSD, whereas other psychological measures did not significantly change from Post to Follow-up (see Supplementary Table S1). In terms of rGMV, bilateral and medial OFC volumes significantly increased (*P*<0.05; small-volume correction, SVC), and right hippocampal volumes significantly decreased (*P*<0.05, SVC) from Pre and Post to Follow-up (Figure 1; Supplementary Table S2), whereas the control subjects did not show above-mentioned rGMV changes between two time points. *Post hoc* correlation analyses revealed that the increase in the volume of the left OFC from Post to Follow-up was significantly correlated with self-esteem scores at Post (*r*=0.43, *P*=0.007; Supplementary Table S3).

The increase in OFC volume identified in some subjects who reported stress indicates that recovery from emotional distress is possible following a stressful event. Previous neuroimaging findings have shown that a reduction in OFC volume is a sign of emotional distress following stressor,^3^ but stress-induced structural and functional alterations in the OFC are reversible.^4^ Although the left OFC volume in our subjects experiencing PTSD symptoms soon after the earthquake decreased in the short term,^3^ the mean OFC volumes increased during this period (Figure 1b), which is consistent with previous findings soon after a disaster.^5^ Moreover, the results provide an initial indication that the increased left OFC volume was caused by higher self-esteem. Given that higher self-esteem is one of the most important traits of resilience in the context of stressful life events,^6^ it is possible that self-esteem is a predictor of increased OFC volume, representing the successful regulation of emotional distress after the earthquake by healthy survivors.

In contrast, stress related to the earthquake may persist even after 1 year. Psychological evaluations at 1 year revealed that even subclinical levels of depression and anxiety levels had not improved from soon after the earthquake. Hippocampal volume reduction is a robust finding in traumatized subjects,^7^ and is observed even in subjects with subclinical depression after a disaster.^5^ Even if the hippocampal volume of young healthy adults were not significantly but slightly reduced as a function of aging (see Supplementary Discussion), post-earthquake stress would accelerate the hippocampal volume reduction because age-related reduction is modified by PTSD and depression.^8^ Together, these findings led us to hypothesize that both prolonged stress and aging affect a reduction in hippocampal volume over time, whereas short-term stress does not reduce hippocampal volume in the period immediately following stressful events such as earthquakes^3^ (see Supplementary Discussion).

The limitations of this study included the absence of psychological assessments and incomplete profiles for the control subjects (see Supplementary Discussion).

Despite these limitations, the present follow-up VBM study found that stressful events had long-lasting effects on various brain structures, suggesting that such changes are influenced by prolonged stress and self-esteem characteristics. Here, it was assumed that structural changes in the brain following stressful life events are not static, but dynamic, throughout one's lifetime. Recently, altered functional and structural connectivity, including in regions adjacent to the OFC and hippocampus as well as in the insula, basal ganglia and parietal lobe,^9,10^ have been reported soon after a disaster. Therefore, further longitudinal investigations using multimodal approaches are necessary to examine whether the stress-induced alterations in brain structure are reversible (see Supplementary Discussion).

## Figures and Tables

**Figure 1:**
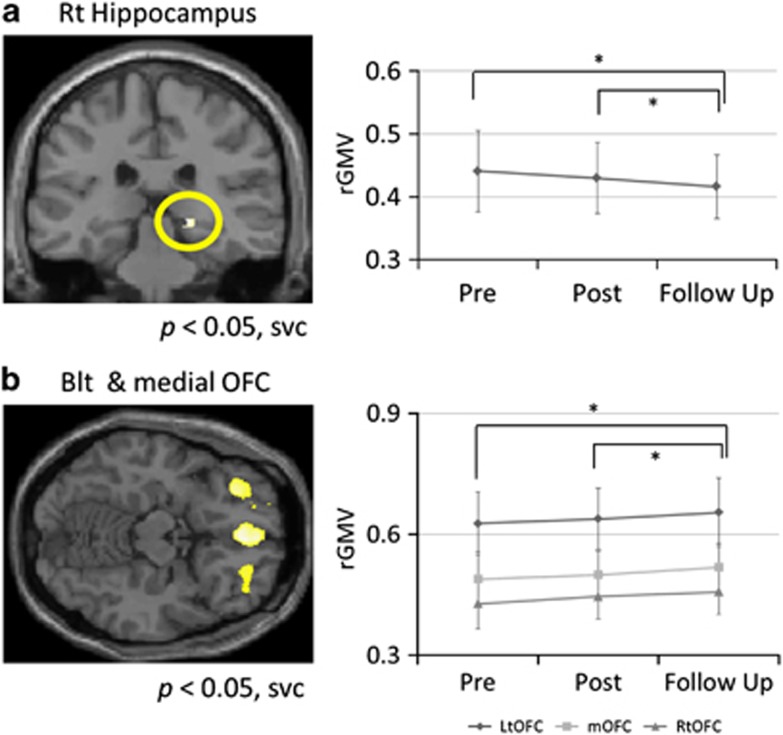
(**a**) Right hippocampal volumes significantly decreased from Pre to Post and from Post to Follow-up and (**b**) bilateral and medial orbitofrontal cortex (OFC) volumes significantly increased from Pre to Post and from Post to Follow-up. These regional grey matter volume (rGMV) changes are illustrated by the plots on the right side, where vertical axes represent rGMV at peak voxels in each cluster, and horizontal axes indicate time periods. Error bars represent s.d. values. Blt, biltateral; Lt, left; Rt, right.
