# Supplementary material for: Resilience after 3/11: structural brain changes 1 year after the Japanese earthquake
Source: Mol Psychiatry. 2014 Apr 29;20(5):553–4. doi: 10.1038/mp.2014.28 (PMC4419148; doi:10.1038/mp.2014.28)
Supplement: Supplementary Information [file mp201428x1.doc]

**Supplementary Information**

**Materials and Methods**

**Subjects**

This study included 37 subjects (M/F = 28/9, age = 21.0 ± 1.6 years) from a total 42 subjects who had participated in a previous investigation by our lab^3^. Each participant was screened for neuropsychiatric disorders using the Mini International Neuropsychiatric Interview (M.I.N.I.) ^11, 12^ and underwent a structural MRI 1 year after the Japanese Earthquake. The M.I.N.I. confirmed that none of the participants had a history of psychiatric illness, including PTSD, and moreover, no participants were exposed to life-threatening experiences during the earthquake or tsunami. Handedness was assessed using the Edinburgh Handedness Inventory^13^. In accordance with the Declaration of Helsinki^14^, before participating in the current study, all participants signed written informed consent forms, which examined the possible effects of psychological trauma on brain structure. The current study was approved by the Ethics Committee of Tohoku University Graduate School of Medicine.

We also analyzed longitudinal brain structural MRI data from our MRI database that were obtained from 11 normal subjects (M/F = 7/4, age = 20.2 ± 1.0 years) before the earthquake. These individuals were treated as a control group, as they had not experienced the earthquake. All control subjects had participated in at least two MRI experiments during over the 300 days before the earthquake and agreed to have their data re-analyzed for future studies. Additionally, control subjects had no history of traumatic brain injury or psychiatric disorders and were not taking medications for psychiatric symptoms according to a self-report questionnaire.

**Psychological evaluations**

All participants were interviewed by trained psychologists using the Japanese version of the Clinician-Administered PTSD Scale (CAPS) structured interview^15, 16^. Confirming the M.I.N.I., none of the subjects was diagnosed with PTSD. We also assessed the subjects’ psychological characteristics, including anxiety, depression, posttraumatic growth, and self-esteem using the State–Trait Anxiety Inventory (STAI) ^17, 18^Center for Epidemiologic Studies Depression Scale (CES-D) ^19, 20^ the Japanese version of the Posttraumatic Growth Inventory (PTGI-J) ^21, 22^ and the Japanese version of the Rosenberg Self-Esteem Scale^23, 24^, respectively. Posttraumatic growth refers to positive changes in oneself due to a struggle against a traumatic event, even though traumatic events often produce negative effects such as PTSD^21^. Higher self-esteem is one of the most important traits for resilience in the face of stressful life events^6^. All psychological measurements were evaluated only after the earthquake.

**Image acquisition**

All magnetic resonance imaging (MRI) data acquisition was conducted using a 3-T Philips Intera Achieva scanner. Using an MP-RAGE sequence, high-resolution T1-weighted structural images (240 × 240 matrix, repetition time = 6.5 ms, echo time = 3 ms, field of view = 24 cm, 162 slices, 1.0-mm slice thickness) of the brain were collected.

**VBM analyses**

To investigate long-lasting structural changes in the brain following the stressful event of an earthquake, VBM was utilized. All VBM analyses were essentially the same as in our previous investigation^3^. First, post-earthquake images were co-registered with pre-earthquake images for each subject on statistical parametric mapping software (SPM2). Preprocessing of the morphological data was performed using an extension of SPM2 software, VBM2^25^, for which default parameter settings were used.

To reduce scanner-specific bias, a customized gray matter anatomical template was created from the pre-earthquake data of all participants included in this study. Next, the T1-weighted structural images for each subject were segmented into gray and white matter partitions using the new gray and white matter prior-probability maps. The resulting images included the extracted gray and white matter partitions in the native space, for which the gray matter partition was then normalized to the new gray matter probability map. The normalization parameters determined from this initial step were then applied to the native T1-weighted structural images and segmented into gray and white matter partitions. The volumes of global gray matter, white matter, and cerebrospinal fluid (CSF) space were calculated using segmented and modulated images by adding a value derived from the voxel volume and multiplied by the value of each voxel. To facilitate optimal segmentation, normalization parameters were estimated using a previously reported protocol^26^. Additionally, a correction was performed for volume changes (modulation) by modulating each voxel with the Jacobian determinants derived from the spatial normalization to test for regional differences in the absolute amount of gray matter^27^.

All images were subsequently smoothed by convolving them with an isotropic Gaussian kernel of 8 mm full-width at half-maximum. Finally, the signal change in rGMV between pre- and post-earthquake images was calculated at each voxel for each participant. Only voxels that showed gray matter volume values >0.10 in pre-earthquake, post-earthquake, and follow-up images were included to avoid possible partial volume effects around the borders between gray matter and white matter and between gray matter and CSF. The resulting maps representing the rGMV before the earthquake (Pre), 3–4 months after the earthquake (Post), and 1 year after the earthquake (Follow-up) were forwarded to the group-level analysis described below.

**Statistical analyses**

Differences in rGMV before the earthquake (Pre), 3–4 months after the earthquake (Post), and 1 year after the earthquake (Follow-up) were assessed using an analysis of covariance (ANCOVA) model on SPM5. The analysis was performed using sex and the period between MRI acquisition and the earthquake as additional covariates. Long-lasting increases and decreases in rGMV were detected by testing the conjunction null hypotheses using conjunction analyses: Pre < Follow-up and Post < Follow-up; Pre > Follow-up and Post > Follow-up, respectively. A small volume correction (SVC)^28^ was applied to each region of interest (ROI) based on the hypotheses (amygdala, hippocampus, ACC, and OFC in each hemisphere) ^3, 29-36^ from the WFU_PickAtlas (<http://fmri.wfubmc.edu/software/PickAtlas>) ^37, 38^ and the Anatomical Automatic Labeling Region of Interest package^39^. The significance level was set at *P* = 0.05, corrected for multiple comparisons (voxel-level family-wise error). Subsequently, we examined data from the control group for GMV changes between the two time points, treating sex and interval between MRI sessions as additional covariates. Finally, to verify the relationship between any structural changes and psychological measures such as self-esteem and posttraumatic growth, *post hoc* correlation analyses were performed employing Follow-up – Post rGMV at peak voxels in each cluster and the total scores of the Rosenberg Self-Esteem Scale and the PTGI-J in Post.

**Supplementary Discussion**

**Hippocampal volume reduction**

Our findings reflect a significant reduction in the hippocampal volume of young adults who had experienced the earthquake, whereas no such reduction was observed in control subjects, who had not experienced the earthquake. Although elderly individuals show robust reductions in hippocampal volume due to aging, ^8^ this is not the case for young individuals; thus, our results are congruent with those of previous studies.

Previous studies have reported that the hippocampal volume of healthy adolescents does not decrease^40, 41^. The subjects in these studies were 7–23 years^40^ and 8–30 years, ^41^ which overlap with that of our participants (19–26 years). Additionally, other longitudinal studies found that the hippocampal volume of young adults did not decrease. Wood et al. reported that 26 normal control subjects (23.8 ± 7.9 yr) showed no significant longitudinal changes in gray matter, including hippocampal, volume after an average interval of 2.2 years^42^. Moreover, Colcombe et al. found no significant longitudinal changes in gray matter, including hippocampal, volume of 20 young control subject (18–30 yr) who did not receive an intervention during their 6-month aerobic exercise training study^43^. Other recent studies of longitudinal hippocampal volume changes led us to infer similar conclusions. Draganski et al. investigated 38 medical students (24 ± 2.3 yr) before and after medical examinations and found that longitudinal hippocampal volume increased over time^44^. Given these findings, we assumed that our subjects, who were also college students who took periodic examinations between the pre- and follow-up periods, would not have shown reduced hippocampal volume if they had not experienced the earthquake during the period under study. Additionally, Woollett et al. found no significant longitudinal changes in hippocampal volume during an average of 32.8 months in 31 control subjects who did not receive an intervention (35 ± 8.99 yr)^45^. Although these subjects were older than those in our study, the previous findings allow us to infer that the hippocampal volume of younger subjects does not decrease within a few years.

In contrast, other longitudinal studies suggest that the gray matter in the temporal lobe does not reach its maximum volume until 16.5 years of age in males and 16.7 years of age in females and that the volume slightly declines thereafter^46^. In fact, subjects in their 20s seemed to show reduced hippocampal volume in another longitudinal study^47^.

Thus, although the hippocampal volume of young adults declines slightly as a function of age, statistically significant reductions are unlikely to occur in a few years in young healthy adults. Additionally, hippocampal volume reduction due to stress is a robust finding,^7^ even in young subjects.^48, 49^ We assumed that both post-earthquake stress and aging contribute to a reduction in hippocampal volume over time.

**Limitations**

Our analysis of the data from control subjects was affected by a few limitations. First, data on psychological issues, such as anxiety and depression, were not available from control subjects. As they had reported no history of psychiatric disorders and no medications for psychiatric symptoms, we assumed their anxiety and depression levels were within normal limits. Second, the profiles of control subjects were not well matched with those of target subjects. Actually, the sample size of control subjects was too small to provide the results for the group X time interaction, which would show that the changes in brain volume in the target group were indeed different from the control group in a strict manner ^50^. However, we supposed the absence of significant hippocampal volume changes in the control group and significant hippocampal volume reduction in the target group partially support the notion that post-earthquake stress would accelerate the hippocampal volume reduction. In addition, the numbers of male and female subjects were not well controlled, and the intervals between the first and second scans of the control subjects were marginally significantly shorter than those of the target subjects (control: 420 ± 108 days, target: 502 ± 144 days, *p* = 0.055, two-sample *t*-test). This was an inevitable limitation because the control subjects were selected from among previously tested subjects with multiple datasets collected at longer intervals. We performed a comparison analysis using sex and intervals as covariates to address this issue.

**Future directions**

Further investigations using multimodal neuroimaging approaches are essential not only to clarify the short- and long-term effects of stressful events on the brain but also to discriminate between survivors with and without PTSD symptoms and between survivors who will and will not develop PTSD. Recent investigations soon after a disaster using multimodal neuroimaging methods, such as structural MRI including diffusion tensor imaging and resting-state functional MRI, revealed lower GMV in the bilateral insula, hippocampus, left caudate, and putamen; greater GMV in the bilateral orbitofrontal cortex and the parietal lobe;^5^ decreased structural connectivity in the right prefrontal lobe, the right parahippocampus, the bilateral basal ganglia, and the bilateral parietal lobe;^10^ and increased functional connectivity in the left prefrontal cortex, the left precentral gyrus, the bilateral insula and caudate, and the left putamen.^9^ These findings indicate that not only the fronto-limbic network, which has been traditionally associated with the psychopathology of PTSD, but also other brain regions are involved in the psychopathology of survivors soon after a disaster. Indeed, recent investigations have revealed that neuroimages from the parietal and occipital regions were good predictors of PTSD symptoms^51^ and clinical PTSD.^52^ In general, we believe that multimodal neuroimaging approaches may contribute to the development of effective means of predicting PTSD.

**Supplemental references**

11. Sheehan DV, Lecrubier Y, Sheehan KH, Amorim P, Janavs J, Weiller E *et al.* *J Clin Psychiatry* 1998; **59** (Suppl 20)**:** 22-33;quiz 34-57.

12. Otsubo T, Tanaka K, Koda R, Shinoda J, Sano N, Tanaka S *et al.* *Psychiatry Clin Neurosci* 2005; **59**(5)**:** 517-526.

13. Oldfield RC. *Neuropsychologia* 1971; **9**(1)**:** 97-113.

14. *Law Med Health Care* 1991; **19**(3-4)**:** 264-265.

15. Blake DD, Weathers FW, Nagy LM, Kaloupek DG, Gusman FD, Charney DS *et al.* *J Trauma Stress* 1995; **8**(1)**:** 75-90.

16. Asukai N, Nishizono-Maher A. Tokyo Institute of Psychiatry: Tokyo, 1998.

17. Spielberger C, Gorsuch R, Lushene R, Vagg P, Jacobs G. Consulting Psychologists Press: Palo Alto, CA, 1983.

18. Nakazato K, Mizuguchi T. Sankyoubou Corp.: Kyoto, 1982.

19. Radloff L. *Applied Psychological Measurement* 1977; **1:** 385-401.

20. Shima S, Shikano T, Kitamura T, Asai M. *Japanese Journal of Clinical Psychiatry* 1985; **27:** 717-723.

21. Tedeschi RG, Calhoun LG. *J Trauma Stress* 1996; **9**(3)**:** 455-471.

22. Taku K, Calhoun LG, Tedeschi RG, Gil-Rivas V, Kilmer RP, Cann A. *Anxiety, stress, and coping* 2007; **20**(4)**:** 353-367.

23. Rosenberg M. Princeton University Press: Princeton, NJ, 1965.

24. Yamamoto M, Matsui Y, Yamanari Y. *Japanese Journal of Educational Psychology* 1982; **30**(1)**:** 64-68.

25. VBM Toolbox for SPM2, VBM Toolbox for SPM5. http://dbm.neuro.uni-jena.de/vbm/. 2007, Accessed Date Accessed 2007 Accessed.

26. Good CD, Johnsrude IS, Ashburner J, Henson RN, Friston KJ, Frackowiak RS. *Neuroimage* 2001; **14**(1 Pt 1)**:** 21-36.

27. Ashburner J, Friston KJ. *Neuroimage* 2000; **11**(6 Pt 1)**:** 805-821.

28. Worsley KJ, Marrett S, Neelin P, Vandal AC, Friston KJ, Evans AC. *Hum Brain Mapp* 1996; **4**(1)**:** 58-73.

29. Karl A, Schaefer M, Malta LS, Dorfel D, Rohleder N, Werner A. *Neurosci Biobehav Rev* 2006; **30**(7)**:** 1004-1031.

30. Yamasue H, Kasai K, Iwanami A, Ohtani T, Yamada H, Abe O *et al.* *Proc Natl Acad Sci U S A* 2003; **100**(15)**:** 9039-9043.

31. Kasai K, Yamasue H, Gilbertson MW, Shenton ME, Rauch SL, Pitman RK. *Biol Psychiatry* 2008; **63**(6)**:** 550-556.

32. Hakamata Y, Matsuoka Y, Inagaki M, Nagamine M, Hara E, Imoto S *et al.* *Neurosci Res* 2007; **59**(4)**:** 383-389.

33. Woodward SH, Schaer M, Kaloupek DG, Cediel L, Eliez S. *Arch Gen Psychiatry* 2009; **66**(12)**:** 1373-1382.

34. Woodward SH, Kaloupek DG, Streeter CC, Martinez C, Schaer M, Eliez S. *Biol Psychiatry* 2006; **59**(7)**:** 582-587.

35. Eckart C, Stoppel C, Kaufmann J, Tempelmann C, Hinrichs H, Elbert T *et al.* *J Psychiatry Neurosci* 2011; **36**(3)**:** 176-186.

36. Chen S, Xia W, Li L, Liu J, He Z, Zhang Z *et al.* *Psychiatry Res* 2006; **146**(1)**:** 65-72.

37. Lancaster JL, Woldorff MG, Parsons LM, Liotti M, Freitas CS, Rainey L *et al.* *Hum Brain Mapp* 2000; **10**(3)**:** 120-131.

38. Maldjian JA, Laurienti PJ, Kraft RA, Burdette JH. *Neuroimage* 2003; **19**(3)**:** 1233-1239.

39. Tzourio-Mazoyer N, Landeau B, Papathanassiou D, Crivello F, Etard O, Delcroix N *et al.* *Neuroimage* 2002; **15**(1)**:** 273-289.

40. Guo X, Chen C, Chen K, Jin Z, Peng D, Yao L. *Neuroreport* 2007; **18**(9)**:** 875-880.

41. Ostby Y, Tamnes CK, Fjell AM, Westlye LT, Due-Tonnessen P, Walhovd KB. *J Neurosci* 2009; **29**(38)**:** 11772-11782.

42. Wood SJ, Velakoulis D, Smith DJ, Bond D, Stuart GW, McGorry PD *et al.* *Schizophrenia research* 2001; **52**(1-2)**:** 37-46.

43. Colcombe SJ, Erickson KI, Scalf PE, Kim JS, Prakash R, McAuley E *et al.* *The journals of gerontology Series A, Biological sciences and medical sciences* 2006; **61**(11)**:** 1166-1170.

44. Draganski B, Gaser C, Kempermann G, Kuhn HG, Winkler J, Buchel C *et al.* *J Neurosci* 2006; **26**(23)**:** 6314-6317.

45. Woollett K, Maguire EA. *Current biology : CB* 2011; **21**(24)**:** 2109-2114.

46. Giedd JN, Blumenthal J, Jeffries NO, Castellanos FX, Liu H, Zijdenbos A *et al.* *Nat Neurosci* 1999; **2**(10)**:** 861-863.

47. Raz N, Lindenberger U, Rodrigue KM, Kennedy KM, Head D, Williamson A *et al.* *Cereb Cortex* 2005; **15**(11)**:** 1676-1689.

48. Carrion VG, Weems CF, Reiss AL. *Pediatrics* 2007; **119**(3)**:** 509-516.

49. Papagni SA, Benetti S, Arulanantham S, McCrory E, McGuire P, Mechelli A. *Stress* 2011; **14**(2)**:** 227-232.

50. Nieuwenhuis S, Forstmann BU, Wagenmakers EJ. *Nat Neurosci* 2011; **14**(9)**:** 1105-1107.

51. Gong Q, Li L, Du M, Pettersson-Yeo W, Crossley N, Yang X *et al.* *Neuropsychopharmacology : official publication of the American College of Neuropsychopharmacology* 2013.

52. Gong Q, Li L, Tognin S, Wu Q, Pettersson-Yeo W, Lui S *et al.* *Psychol Med* 2013**:** 1-9.

**Supplemental Table S1. Psychological measures**

|  | Post | Follow-up | *p*-value |
| --- | --- | --- | --- |
| CAPS (Total) | 5.2 ± 9.6 | 1.6 ± 2.9 | 0.03 |
| CES-D score | 12.1 ± 10.6 | 11.8 ± 10.6 | n.s. |
| STAI scores |  |  |  |
| State | 42.3 ± 11.8 | 39.9 ± 11.8 | n.s. |
| Trait | 43.1 ± 10.4 | 43.8 ± 12.0 | n.s. |
| Self-esteem | 33.5 ± 7.9 | 33.5 ± 8.3 | n.s. |
| PTGI-J (Total) | 34.7 ± 17.9 | 33.9 ± 17.9 | n.s. |

Values are shown as means ± standard deviations.

CAPS, the Clinician-Administered PTSD scale; CES-D, Center for Epidemiologic Studies Depression; STAI, State–Trait Anxiety Inventory; PTGI-J, Japanese version of the Posttraumatic Growth Inventory

**Supplemental Table S2. MNI coordinates, voxel sizes, *z*-scores, and *P*-values for the results of SPM analyses**

| Brain region | MNI coordinates | | | κ (voxels) | *z*-scores | *P*-values (SVC) |
| --- | --- | --- | --- | --- | --- | --- |
|  | x | y | z |  |  |  |
| Decreased |  |  |  |  |  |  |
| Rt Hippocampus | 19 | -28 | -6 | 171 | 3.55 | <0.05 |
| Increased |  |  |  |  |  |  |
| Lt lateral OFC | -32 | 35 | -14 | 1508 | 3.99 | <0.05 |
| Blt medial OFC | 2 | 42 | -14 | 1833 | 4.44 | <0.05 |
| Rt lateral OFC | 20 | 44 | -19 | 1479 | 4.23 | <0.05 |

MNI, Montreal Neurological Institute; Blt, biltateral; Rt, right; Lt, left; OFC, orbitofrontal cortex

**Supplemental Table S3. Correlation coefficients between increased OFC volumes and psychological measures such as Self-esteem and PTGI-J scores**

| Brain areas | Pearson’s *r*  (*p*-values) | |
| --- | --- | --- |
|  | Self-esteem | PTGI-J |
| Lt lateral OFC | 0.43*  (0.007) | -0.19  (0.26) |
| Blt medial OFC | 0.25  (0.14) | 0.03  (0.87) |
| Rt lateral OFC | 0.27  (0.11) | -0.16  (0.36) |

PTGI-J, Japanese version of the Posttraumatic Growth Inventory; Blt, biltateral; Rt, right; Lt, left; OFC, orbitofrontal cortex, * *p* < 0.05
